# The Mortality from Influenza

**Published:** 1900-02-10

**Authors:** 


### THE MORTALITY FROM INFLUENZA.

The Registrar-General, unfortunately, has not yet added influenza to his list of "principal zymotic diseases," so that the Quarterly Return just issued does not contain any direct statement with regard to the mortality occasioned by this malady during the last three months of 1899. Some notion of its extent may, nevertheless, be gleaned from the general fact that the mortality from the official "zymotic diseases," measles, diphtheria, diarrhœa, "fever," whooping-cough, scarlet fever, and small-pox, was 0·22 per 1,000 below the mean proportion in the fourth quarters of the ten preceding years. On the other hand, the total mortality for the quarter of persons over 60 years of age showed an increase of no less than 14·2 per cent. over that of the corresponding quarters of the last ten years, and reached 108·7 per 1,000 in Bristol, 106·6 in Portsmouth, an average of 90·7 per 1,000 in 33 great towns, and of 79·1 per 1,000 in 67 large towns of the next magnitude. No specific information as to the causes of this great increase of mortality among persons advanced in life is contained in the Quarterly Return, and these causes will probably be ultimately divided among a variety of such headings as bronchitis, pneumonia, or heart failure. There can be no possible doubt, however, that the deaths must have been mainly attributable either to influenza or to its consequences, and that the methods of defence against so destructive a disease should receive the very earnest attention of the medical profession.

